# Tuberculosis in Juvenile Idiopathic arthritis under Anti-TNF therapy

**DOI:** 10.1186/1546-0096-9-S1-P72

**Published:** 2011-09-14

**Authors:** G Espada, J Giupponi, C Cerqueiro

**Affiliations:** 1 Rheumatology and Tisiology Sections, Hospital de Niños, Dr Ricardo Gutierrez, Buenos Aires, Argentina

## 

Tuberculosis (TB) is still a prevalent disease, one third of the population is infected by *mycobacterium tuberculosis (MT)*.Treatment with anti TNF therapies appears to increase the risk for developing TB

## Aim

To study the occurrence of latent TB (LTB) or tuberculosis disease (TBD) in children with JIA under anti TNF therapy.

## Methods

Clinical records from pts with JIA ( ILARÂ´01) under anti-TNF therapy were reviewed, focusing on TB screening prior to biologicals , PPD conversion or TBD diagnosiss.LTB was considered as a PPD (TST) positive (≥ 5 mm), in the abscense of radiological manifestations of TB.

## Results

105 JIA pts received one or more anti TNF drugs during the observation. Four of them developed LTB and 3 others TBD.

**Table:** 

	Gender/ age( ys)	Dx	PPD (mm)*	TBDisease	antiTNF	Time under 1st Biologic	TB tx	2nd Biological Tx
Pt 1	M / 18	pJIA	18	no	ETN	24 m	INH	No
Pt 2	F/ 20	oJIA	12	no	ADA	24 m	INH	No
Pt 3	F / 7	pJIA	6	no	ETN	11 m	INH	INF
Pt 4	M / 13	sJIA	13	no	IFX	1 m	INH	No
Pt 5	F / 14	pJIA	10	Pulmonar involvement	IFX	6 m	4 drugs	ETN
Pt 6	F / 17	pJIA	13	Pulmonar involvement	IFX	24 m	4 drugs	No
Pt 7	F / 12	pJIA	13	Pulmonar involvement	ETN	30 m	4 drugs	ETN

No extrapulmonar TB or miliary forms were observed. All patients were PPD negative prior to biological agents

## Conclusions

No significative difference was found between specific anti TNF and the occurrence of LTB or TBD. In 4 pts, the infectious event occurred after 2 ys of exposure and in two earlier as described in the literature. This data highlights the importance of TB surveillance prior to and during anti-TNF therapy.

